
JOURNAL INFORMATION
==============================
NLM Title Abbreviation: Med Chir Rev
NLM Journal ID: 0034617

The Medico-Chirurgical Review

Publisher: Biomedical Journal Digitization Project

ARTICLE INFORMATION
==============================
PMCID: PMC4966257
PMID: 29917628
Article version: 1
Subjects: Reviews

Pathological and Surgical Observations Relative to Injuries of the Brain

Print publication date: 1828 Oct 1
Volume: 9
Issue: 18
First page: 409
Last page: 432
1828 Sep
License: This work is free of known copyright restrictions. For more information, please see PMC Back Issue Digitization.
License URL: https://creativecommons.org/publicdomain/mark/1.0/

==============================

### XIV.

*Pathological and Surgical Observations relating to Injuries of the Brain.* By B. C. BRODIE, F.R.S. and Surgeon to St. George's Hospital.

[Medico-Chirurgical Transactions, Vol. XIV.]

THE history of the world has been rightly denominated the history of a ceaseless train of revolutions, physical, political, and moral. Ever changing, ever changed, the features and creatures of yesterday are vanishing, or vanished, to-day: and man, proud man, the architect alike of cities and systems, the founder of nations, and inventor of sciences, sinks by the law of nature to the dust, that dust to which his cities, his systems, and too often his sciences, follow him. Creeds of physic, like religions, "take their turn;" Hippocratists give way to Brownonians—Brownonians to disciples of Broussais—the Humorists have been routed by the Solidists, and at present, there appears to be some probability of the Solidists being again dispossessed of their sway by the Humorists! Whilst physic, as a whole, is submitting to changes, its fractional parts, the cantons or divisions, as it were, of the republic, are also the prey to revolutions. Take, for instance, the head, and the treatment of the injuries to which it is subject. What varieties of opinions and practice have existed—what doubt and uncertainty unhappily exist! In the days of Mr. Pott, the trephine was in constant requisition, whilst, since the publication of the work of Mr. Abernethy, it has almost grown rusty in its case!

Though theories, however, are changing, some now in their decline, and some irrecoverably fallen, *facts* still remain. The theorist in medicine may be right or may be wrong, but the patient and industrious collector of facts is conferring an inestimable boon on the profession, and surely, if slowly, contributing to its improvement. The paper, to the analysis of which we are proceeding, is eminently rich in the *matériel* of science, authenticated cases and practical observations. Such a paper as this may excite less éclât than one that is adorned with the sallies of imagination, the refinements of ingenuity, and the touches of sarcasm, but its value will remain, when much of the ephemeral interest of its rival has subsided. The labour in constructing such a paper must be great, especially to one like Mr. Brodie, engaged in the most extensive practice of the day. A theory may be engendered and brought to maturity in the closet, but the observation of almost innumerable cases must have occupied many a wearisome hour in the wards of the hospital and chambers of the sick, whilst the time which is spent in recording them, must inevitably be snatched from the very brief interval of repose, if repose it can be called, that a man at the summit of his profession can enjoy! Of Mr. Brodie's experience, and the practical value of any thing which proceeds from his pen, the public are already so very well aware, that comment on either is totally unnecessary. The greatest advantage we can render the profession, and most flattering compliment we can pay to the author, is not by indulging in laboured panegyric, but giving a full and impartial account of the litera scripta—the paper in question.

Mr. Brodie has considered it necessary to do, what none, we are sure, will ever deem it necessary to do *for* him, viz. apologize for writing on the subject.

"If any apology be necessary for this undertaking, I may remark, that I have been led to it by the great importance of the subject, and also by this consideration, that, although much information may lie, as it were, scattered throughout the mass of surgical literature, no practical writer, as far as I know, has attempted to make such a collection and arrangement of facts as will enable the surgical student to take a distinct and connected view of all the parts of this curious and interesting inquiry. The present paper contains only a part of the observations which I have to offer, namely, those which relate to the first or immediate consequences of the injury. Should these be favourably received, I shall venture, on a future occasion, to communicate to the Society a second series of observations relating to those more remote consequences which are connected with inflammation of the brain and its membranes, or which arise after inflammation has subsided." 2.

The paper is systematically divided into sections, each section embracing the consideration of some particular point of importance. The subject of the first is:—

### The immediate Effects of Injuries of the Head, as indicated by Dissection.

Though various, and variously complicated, these admit of being classed under the following heads:—1mo. Simple contusion of the scalp, with extravasation of blood between it and the tendon of the occipito-frontalis, or between the latter and the pericranium, or between the pericranium and the bone. The sensation of depression of bone which is given to the fingers by the margin of extravasated blood, is familiar to all. 2do. The scalp may be lacerated, or the pericranium itself may be torn from the bone; the latter the more serious injury of the two. 3tio. The small vessels connecting the dura mater to the bone may be ruptured at the part where the injury is inflicted, and the dura mater separated to a greater or less extent. This is not an unfrequent occurrence, and at times the separation is very extensive. In the case of a boy at St. George's Hospital, who had fallen from a height of fifty feet, besides a large extravasation on the inferior surface of the brain, the dura mater was every where separated from the bone, except at the basis of the cranium, and the outside of the membrane had a sloughy appearance. 4to. There are many varieties of fracture. In most instances it occurs at the upper part of the skull, whilst that at the basis is always the consequence of violent injury, and generally fatal, because it is almost invariably accompanied with lesion of other more important parts.

With regard to what the French have denominated the *contre-coup*, Mr.

Brodie does not absolutely deny its occurrence, but remarks that his own experience very nearly coincides with Mr. Earle's, who has not known a fracture of this kind take place, except where the blow appears to have so operated, as to impel the occiput with force against the atlas, the line of fracture passing through the former bone, where it rests upon the latter. Some cases of contre-coup are recorded in the Memoirs of the French Academy of Surgery, but in many of them, there seems to be no sufficient evidence that the fracture did not really arise from a second blow, inflicted on another part of the head. In the cases adduced by Mr. Bell, the fracture extended across the occiput, in one case passing through, and in the other case passing close to the foramen magnum.

In all cases of fracture of the cranium, with depression, the inner table is broken more extensively than the outer, sometimes, indeed, distinctly depressed, whilst scarcely a fissure exists in the latter. Instances are related by Tulpius, Parey, and Dr. Hennen, in his Treatise on Military Surgery, where the outer table was sound, whilst the inner was extensively fissured, broken into splinters, or actually driven, for more than half an inch, into the membranes of the brain.

5to. The skull of a child is frequently indented after the infliction of a blow, and restored in a few days to its natural level, without the assistance of art. Mr. Brodie supposes that the earthy part of the bone, in these cases, gives way, while the animal portion is entire, preventing a fracture, or actual solution of continuity, and allowing the pulsations of the brain to remove the depression.

6to. The disjunction of the sutures, which is rare, and can of course only happen in early life, when the sutures are not yet consolidated, is always to be reckoned peculiarly dangerous, as the force that must produce it is so great, as, in all probability, to cause other and serious injuries.

7mo. Extravasations of blood may occur between the bone and the dura mater, from the rupture of the small vessels by which they are connected, or laceration of the branches or the trunk of the arteria meningea media. Mr. Brodie's experience confirms the opinion advanced by Mr. Abernethy, that dangerous extravasation is never produced, except where the latter has been wounded, an accident which seldom takes place, except in combination with fracture of the bony canal which protects it. Extravasation may also occur within the dura mater, and here it is generally collected between the latter and the tunica arachnoides; sometimes, but rarely, in the ventricles; at others, in the substance of the brain, or the cellular texture between the arachnoid and pia mater. Large extravasations more frequently are found on the basis than the superior aspect of the brain, in consequence of the irregularities on the surface of the former, the hæmorrhage being usually dependent on a rupture of the substance of the cerebrum or cerebellum. As the laceration of the brain but seldom occurs at the spot when the blow is received, it may be said with propriety to afford an example of the *contre-coup*. Wounds of the sinuses, though bleeding profusely, when the blood can escape by a free opening in the bone, are not, in Mr. Brodie's opinion very dangerous, the hæmorrhage being readily suppressed by a very slight pressure. The bleeding from the ear which occasionally occurs, especially where the basis of the cranium is fractured, must in most instances arise from a laceration of the lateral sinus, as it lies behind the external meatus and the petrous portion of the temporal bone. In a case in which there was bleeding from the ear and the nostrils, it was found, on dissection, to proceed from a laceration of the cavernous sinus. There may take place all descriptions of wounds of the brain and its membranes, lacerated, punctured, and incised; with loss of substance or without it; complicated or not with the effects of contusion already described.

### SECT. II.—CONCUSSION OF THE BRAIN.

Wounds, lacerations, and compression of the brain may, indeed generally must, impair or destroy the functions of the organ, but still it is evident that other injuries, as fissures, disjunction of the sutures, or separation of the pericranium or dura mater, can scarcely of themselves, produce such effects in the first instance. A man gets a blow upon the head, becomes insensible, and dies. On examination, the brain and its membranes are sound, and this stunning or concussion does not seem to depend on any cognizable derangement of structure.

"We are not however justified in the conclusion that there is therefore in reality no organic injury. It is difficult to conceive in what other manner concussion of the brain can operate so as to produce the effects which it is known to produce; and if we consider that the ultimate structure of the brain is on so minute a scale that our senses are incapable of detecting it, it is evident that there may be changes and alterations of structure, which our senses are incapable of detecting also. The speedy subsiding of the symptoms of concussion does not contradict this opinion. A deep incised wound in other parts of the body may, under certain circumstances, be completely and firmly united in the space of twenty-four hours; and it is easy to suppose that the effects of a much slighter injury may be repaired in a still shorter space of time." 14.

The grades and the duration of concussion are various. The patient may recover in the course of a few minutes, or the total insensibility be succeeded by a state, in which the sensibility is impaired, but not destroyed.

"The patient is not affected by ordinary impressions, but if spoken to in a loud tone of voice, he will shift his position, and answer in a peevish manner. Sometimes he is in a state of imperfect delirium, talking in an incoherent and rambling manner, as if intoxicated. The pupils contract on exposure to light, and are sometimes more contracted than under ordinary circumstances. There is no paralysis. The respiration in the great majority of cases is performed easily and naturally; in a few instances only it is laboured, and approaching to being stertorous." 14.

These symptoms may subside in a very few hours, or continue for three or four days, the sensibility in the latter case frequently returning for a time, but only again to be speedily lost. Where inflammation is the sequence of concussion, there may be a considerable interval of sense, or the symptoms of the latter may gradually and imperceptibly be converted into those of the former.

Concussion of the brain is mostly accompanied with head-ache, sometimes but transient, at others intense, and remaining for days " a solitary symptom, after all other symptoms are vanished." Sickness and vomiting are early symptoms and generally soon pass away. The patient of course retains no recollection of aught that occurred during the state of concussion or insensibility, but frequently the memory is affected to a greater extent. The case of the Welsh labourer in St. Thomas's Hospital, who forgot all his English, but remembered the Welsh, and the parallel instance of the German sugar-baker, recorded by Sir Astley Cooper are celebrated and amusing examples of the fact. Two cases where the patients lost all recollection of what they were doing immediately antecedent to the accident, are recorded by Mr. Brodie, and allusion is made to the still more remarkable case of Dessault, where, after a blow upon the head, the man could remember *recent* events only, but afterwards experienced a change, his memory failing him in these, and merely retaining what had happened in his childhood. Independent as the action of the heart appears, to a certain extent, to be of the influence of the brain, the circulation in concussion is generally more or less affected. The pulse is intermitting, irregular, feeble, perhaps scarcely perceptible, whilst the patient is in a condition approaching to syncope, a condition that may obtain for some minutes, or even for four or five hours. In those cases, they are rare, in which concussion proves fatal, the immediate cause of death appears to be this disturbance of the action of the heart. The re-action, which is generally felt, never comes, or coming is imperfect—the feebleness and irregularity of the pulse are increased—the extremities grow cold—the heart fails entirely—and the patient expires.

### SECT. III.—COMPRESSION OF THE BRAIN.

That the functions of the brain are impaired when the cavity of the cranium is by any cause suddenly diminished, there is no dispute whatever, although the physiological rationale of the phenomena is the source of a great deal of difference of opinion. Mr. Brodie agrees with Mr. Bell in believing, that the substance of the brain is incompressible, and, therefore, that what is called " compression" of the brain, operates simply by lessening the diameter of its blood-vessels and preventing that supply of arterial blood which is necessary for the proper performance of its functions.

" It may, however, be urged on the other hand, first, that in some cases symptoms similar to those which arise from compression, take place where there is a preternatural determination of blood to the head; where the vessels instead of being empty are actually overloaded; and that in these cases the symptoms are relieved by drawing blood from the jugular vein, or from the veins of the arm; as if the pressure occasioned by too much blood in the vessels was productive of nearly the same effects on the brain, with that arising from blood in a state of extravasation: secondly, that, although we admit the substance of the brain to be incapable of being compressed into a smaller compass, yet that the effect of all pressure on it must be, and is, to alter the position and relative situation of the delicate fibres of which its minute structure is composed, and that we need seek no further explanation of the symptoms which are met with in these cases." 20.

We certainly think that the explanation of the symptoms of compression must be sought for elsewhere, than the paucity of arterial blood in the vessels of the brain. In syncope from hæmorrhage, the brain is not duly supplied by scarlet blood, but a state is produced, which has little analogy as regards the phenomena it presents, with that which obtains in cases of compression.

Although, for the most part, an extensive depression is attended with more danger than a slight one, a large extravasation than a small, the production of symptoms is often dissimilar in different cases, though the injury, cæteris paribus, is the same. Pressure, on the whole, is more dangerous at the base than the upper part of the brain, and it seems to Mr. Brodie, that a given quantity of blood between the tunica arachnoides and pia mater, produces worse symptoms than when it is collected in a mass, and occasions less general pressure.

After making these preliminary observations, Mr. Brodie proceeds to consider the particular symptoms of compression. That the *pain in the head* may in some cases depend on the concussion which the brain has received, is very probable, but that sometimes it is wholly and solely occasioned by the pressure on the brain is proved by a case of Mr. Brodie's, where immediately after the elevation of the depression this symptom entirely disappeared. 2. *Insensibility.* This may be either incomplete, the patient being capable of being roused by powerful impressions on his senses, or total and complete. When the cause is depression of bone, the insensibility is developed immediately after the infliction of the injury, but where it depends on extravasation of blood, the extravasation, in many cases, taking place slowly, an interval of time, as an hour for instance, may elapse before it shews itself. Insensibility may depend on concussion in the first instance, in which case the patient may recover, and gradually relapse into the state of insensibility again, as the blood is effused within the cranium. This observation is important as regards the diagnosis of these different species of injury. The insensibility dependent on actual compression is liable to variation, the patient at one time recovering some consciousness, and then again relapsing, whilst blood-letting is followed by increase of sensibility, which departs as the effect of the remedy subsides.

"If these observations be correct, it is evident that there is not any such difference in the character of the insensibility produced by concussion, and that produced by compression of the brain, as will enable us at once, and in all cases, to distinguish these two kinds of injury from each other. Those who are led to take a different view of the subject, may indeed urge, that in some cases there is considerable pressure on the brain, without any symptoms at all; and that when, in a case of fracture and depression of the cranium, or extravasation of blood within the cranium, the patient lies with a partial loss of sense, this is to be attributed not to the actual pressure, but to the concussion of the brain, which the violence inflicted must necessarily have occasioned in a greater or less degree. I might however refer to several cases, to which this explanation cannot be well applied; but a single example will be sufficient. A woman received a blow on the head; after which she was able to walk home, complaining that her head was hurt, and that she had received her death blow. In an hour after the accident, she gradually became insensible. About fourteen hours afterwards she was brought to St. George's Hospital, labouring under symptoms precisely corresponding to those which have been described by Mr. Abernethy, as arising from concussion. These symptoms continued, and even rather abated than increased, until the third day, when an aggravation of them took place, and she expired. On examining the body, eight ounces of blood were found effused underneath the dura mater. The circumstance of there having been no loss of sense in the first instance, and the interval of an hour which elapsed between the period of the accident and that of the occurrence of the symptoms, sufficiently demonstrate that they were the consequence of pressure produced by the hæmorrhage, and not of the concussion." 24.

In some cases, affection of a particular sense or partial insensibility only is observed. An old man who suffered from fracture with depression of one of the parietal bones, though slow in giving answers, was otherwise sensible, save that he was noticed to be totally blind. The trephine was applied, but the symptoms continued, and on the fifth day after the accident, he died. The membranes of the brain were found inflamed, and a fracture extended across the sphenoid bone, the edges of which pressed on the optic nerves immediately behind the orbit, and accounted for the total loss of sight. Cases of numbness, or loss of sensation in a particular part, after symptoms of compression are not very uncommon.

3. *Paralysis.* In cases where sensibility is totally destroyed, paralysis is complete, the bladder becoming distended with urine, and the fæces involuntarily discharged. The muscles of respiration are afterwards affected; the breathing is stertorous, the diaphragm contracts at longer and longer intervals, and finally ceases to act altogether, the paralysis of these muscles constituting in fact the immediate cause of death in ordinary cases of pressure on the brain. Where the loss of sense is imperfect, there are often no marks of paralysis whatever; at other times the patient is affected with hemiplegia, which may even be permanent, although he should otherwise recover. Hemiplegia, however, is infinitely more common after apoplexy than accidental injury to the brain, which would seem to arise from the extravasation, in the former case, being seated in the ventricles or substance of the brain: in the latter, most commonly on the surface. The paralysis may affect only one set of muscles, or even a single one, as in ptosis of the eye-lid. In cases of injury, the hemiplegia is observed on the opposite side of the body, which does not apply to more partial paralytic affections. A gentleman struck the side of his head against a carriage wheel, and produced an ecchymosis of the left cheek and temple, accompanied with bleeding from the ear and paralysis of the muscles supplied by the portio dura. The gentleman recovered in the course of three months, and there cannot be a doubt that the pressure was produced on the portio dura, and not upon the brain. The case is exactly analogous to those which are described by Mr. Bell, and frequently met with in practice. Mr. Brodie has known ptosis of the left upper eye-lid, connected with pressure on the nerve of the third pair, immediately behind the *left* cavernous sinus.

4. *Convulsive Actions of the Muscles.* Slight and partial convulsive twitches are sometimes observed in one side of the body, whilst the other is affected with palsy. In the cases of this kind that have fallen under Mr. Brodie's observation, when pressure has existed on the brain it has always been accompanied with wound or laceration of its substance.

5. *Affections of the Pupils.* These vary extremely, and even under circumstances apparently similar, although, for the most part, the pupils are insensible and motionless—generally dilated, but sometimes contracted. Sometimes, without apparent cause, dilatation gives way to contraction, and the latter again to dilatation. If the pupils were dilated, contraction not unfrequently follows the abstraction of blood, the dilatation returning as the immediate effect of the blood-letting ceases. Reversing the phenomena commonly observed, dilatation has followed an *increase* of light, and contraction its *diminution*—one pupil has been dilated, the other being natural or even contracted; and again, in a fracture with depression above the right superciliary ridge, both pupils were dilated and equally insensible, but regained their sensibility on raising the depression. A pupil may continue insensible to light, without the concurrence of general insensibility, or, indeed, without any loss of vision whatever. This happened in a gentleman, accompanied with ptosis of the right upper eye-lid, and numbness of the right hand, a year nearly elapsing before the pupil recovered its natural condition.

6. *Affection of the Circulation.* The effect of compression on the circulation is not constant, sometimes not present at all. Mr. Abernethy has observed, that intermissions of the pulse are less frequent in compression than concussion, but Mr. Brodie believes that the former affects, for the most part, the action of the heart by causing its contractions to be either less frequent or less forcible than natural. In a child at St. George's with fracture of the parietal bone and neighbouring portions of the temporal and occipital, the pulse at the wrist was barely perceptible; but, at the instant of raising the depression, grew distinct and even strong. In another case, the pulse, which before the operation was forty, rose to sixty in the minute immediately afterwards. It strikes us, that some degree of fallacy exists in these observations on the pulse. The patient, it should be remembered, is undergoing a severe and tedious operation, generally attended with little loss of blood, and exceedingly likely to quicken the action of the heart, independent of the removal of pressure from the brain. In a fatal case of concussion, accompanied with slight extravasation in the substance of the brain, it was necessary to secure some vessel which was wounded by the blow upon the scalp. The pulse rose distinctly during the performance of this slight operation, though, of course, it could have no immediate effect upon the brain, and the patient continued insensible, though struggling indeed, as they commonly do, under the influence of the pain. In another case which we witnessed of compound fracture and depression of the temporal bone, which proved fatal in a very short time, the pulse rose remarkably during the application of the trephine, but it evidently depended on the general excitement.

7. *Sickness and Vomiting.* These can scarcely be ranked amongst the symptoms of compression, being far from uniformly present. Mr. Brodie has known them succeed the elevation of the depression, though the patient shewed no disposition to them previously.

The combination and duration of the symptoms of compression are exceedingly various, one patient dying in three or four hours, and another surviving for several days under circumstances apparently similar. In those who recover, a similar want of uniformity is observed, and in fatal cases the symptoms are not regular in their march, halting at times, and then hurrying on with accelerated pace. Secondary hæmorrhage occurring not unfrequently in other situations, it becomes a very interesting question if it ever takes place in the brain. A middle-aged man having fallen from a cart with his head upon the pavement, was bled by a practitioner, brought to St. George's, and there bled again. On the following day he complained of some head-ache, but was otherwise well, and continued so till the morning of the 4th day from the accident, when he was suddenly seized with the symptoms of apoplexy, and died in half an hour. A thin layer of blood was effused between the pia mater and tunica arachnoides, at the posterior part of the hemispheres of the cerebrum. Besides this, at the lower part of the right anterior lobe, the substance of the brain had been ruptured, and a recent extravasation of blood, amounting to about two ounces and a half was discovered between the arachnoid and dura mater. This is certainly very like secondary hæmorrhage, and probably, as Mr. B. observes, its occurrence would be oftener remarked, were it not for the rigid antiphlogistic regimen which is usually adopted in injuries of the head.

### Sect. IV.—Wounds of the Brain and its Membranes.

Wounds of the dura mater are not productive of serious consequences till the period of inflammation has arrived; whilst those of the arachnoid and pia mater can scarcely take place independently of wounds of the substance of the brain. The physiologist, as he slices off portions of the brain, deprives the subject of his experiments of function after function. The same distinct results are, however, not the consequence of accidental wounds, the complications of which with concussion, extravasation, or depression of bone, are so various and extensive as to prevent the effects of any given injury in a given situation, being calculated with any degree of precision. Two cases are detailed from Morgagni and Dr. Hennen, in one of which a sharp instrument passed between the roof of the orbit and the eye, and pierced through the substance of the cerebrum to within a finger's breadth of the lateral ventricle. In the other, a ramrod entered below the nasal process of the os frontis, and penetrated an inch into the anterior lobe of the cerebrum. This patient, in whom the injury appears to be the slightest, was killed upon the spot, whilst the other presented not a symptom until the third day, when suppuration was established. As a general rule, the functions of the brain are but little impaired, if at all, in the first instance, by penetrating wounds of its substance. A patient, indeed, may go on from day to day, with portions of brain oozing out from an aperture in the cranium, without any affection of the functions of body or mind. Extensive destruction, however, of the brain is generally followed by death, either immediate or occurring in the course of a very few hours. It has seemed to Mr. Brodie, that in other cases of extensive laceration of the brain, independent of the actual insensibility of concussion, "there obtained a confusion of intellect beyond that which concussion usually produces." In many cases there are twitchings of the muscles of the extremities; in one, where the parietal bone was fractured, and splinters driven in upon the cerebrum, the opposite pupil continued preternaturally dilated when the splinters were removed, though nothing but the wound apparently remained. The pulse, as in cases of pressure, is sometimes unnaturally slow, but the symptoms are seldom so urgent, the danger not being immediate, but depending on the violence of the after-inflammation.

### SECT. V.—ON SOME OTHER SYMPTOMS FOLLOWING INJURIES OF THE BRAIN.

The symptoms to which this section is confined shew themselves anterior to the occurrence of inflammation, but present an anomalous character, and cannot be fairly considered under any of the preceding heads. Thus, a middle-aged man was admitted into hospital with the symptoms of concussion, which subsided in one or two hours, but left irremediable deafness. A young woman was stunned by a blow upon the head, and after recovering from its immediate effects, was entirely deprived of the senses of smell and taste, in which state she continued at the end of a month, when Mr. Brodie saw her. Such numbers of cases of this kind are on record, that we deem it unnecessary to follow Mr. Brodie through several very interesting illustrations. These cases are distinguished by the absence of the symptoms of compression of the brain, and are infinitely more difficult to account for satisfactorily than a person at first would imagine.

"It is indeed difficult to conceive that pressure on the brain should exist in so great a degree as completely to destroy an entire class of sensations, and at the same time be so partial as not to affect any other function of the nervous system. On the other hand it is also difficult to regard these as the effects of concussion of the brain: since it is one of the characteristics of concussion to produce no more than a diminution of sensibility, and that diminution, instead of continuing for months or years, is completely relieved in the course of a few days, and probably in a much shorter space of time." 42.

Dr. Hennen gives the history of a patient, who lost his sexual powers after wounds of the occiput, and various instances of a similar description are met with in authors. The sight has been frequently impaired whilst the faculty of hearing remained, and sight has continued whilst speech has been lost. Stammering, we believe, is an occasional consequence of blows upon the head.

In some cases the patient is seized with convulsions, a good deal resembling those of epilepsy, though differing from the latter in not being succeeded by stupor or sleep. Though formidable in appearance, they frequently subside without any unfavourable symptom. In the case of a butcher, who was struck on the head by a beam, venesection was employed without relief, but, after continuing three hours, the convulsions suddenly subsided, leaving him sensible, but affected with pain in the head, which required two or three bleedings in the course of the ensuing week or ten days. In the case of a gentleman, who, in addition to a fracture communicating with the elbow-joint, got a blow upon the head, which occasioned a considerable scalp-wound and fracture of the os frontis, unattended with depression, the convulsions came on in the course of a few minutes, were exceedingly violent, but subsided in about half an hour. The gentleman recovered. In some cases, the patient is affected with convulsions, not immediately after the occurrence of the accident, but after the lapse of several days. In such it must often be difficult to determine to which the convulsions are referrible—the original mischief or subsequent inflammation. A lad received a blow upon the head, insensibility followed, but he moaned when disturbed, the pupils were obedient to the light, and neither stertor nor paralysis were present. The symptoms subsided, no appearance of inflammation came on, but the patient was seized, at the end of five days, with general convulsions. He was cupped without relief, and, in the course of the succeeding twenty-four hours, he had as many as fourteen or fifteen attacks, each lasting from one to three minutes. On the following day the symptoms not indicating bleeding, the patient was prevailed on to swallow some toast and beef-tea, from the commencement of which practice the convulsions abated, and in the course of another day wholly disappeared. This case, as Mr. Brodie observes, seems to prove that the convulsions, at this late or rather secondary period, depended on the original mischief, and not on inflammation. In two of the cases detailed, the other symptoms were probably those of concussion, but Mr. Brodie suspects that more than concussion is required to produce these attacks of convulsions, or, at any rate, that they may be brought on by other causes. Mr. Brodie is led to this opinion by observing in experiments on animals, that a wound on the basis of the brain, which produces extravasation of blood upon its surface, in general gives rise to convulsions, previous to that state of paralysis and stupor which immediately precedes the creature's death. In the next place, the symptoms of concussion are most complete immediately after the infliction of the injury, whilst convulsions do not occur until after a certain lapse of time, when extravasation may possibly have begun to take place. Finally, in a case that occurred to Mr. Keate, when convulsions came on at the end of two days, and persisted in spite of the abstraction of a quantity of blood from the arm, an incision was made through the scalp at the part which was injured—a fracture discovered with trifling depression—the depression removed with a saw—and a coagulum of blood found lying on the dura mater, which having been exposed, the convulsions disappeared and returned no more.

"I have not observed convulsions to take place where there are symptoms indicating the existence of considerable pressure on the brain. The pressure in these cases does not destroy the functions of the brain; it seems to act merely as a cause of irritation, and the operation of it may be compared to that of an exostosis, or other tumor, in producing fits of epilepsy. The circumstance of convulsions taking place after the lapse of some days when they did not take place in the first instance, may probably depend on the brain having been rendered more susceptible by the loss of blood and other methods of depletion to which it was necessary to have recourse for the relief of the more early symptoms." 48.

An occasional consequence of injuries of the head, is furious delirium. Though present in cases apparently of concussion, still we are not sure that no degree of pressure existed on the brain, whilst instances occur in which this condition of sensorium is manifestly combined with depression of bone, or extravasated blood. Some interesting cases are detailed by Mr. B. in illustration. In the first, the delirium subsided on blood being taken from the arm, and the patient remained with slight symptoms only of concussion, from which he completely recovered. In the second case the frontal bone was fractured and depressed. The depression was not raised, as the delirium subsided on blood being taken from the arm, when the patient fell into a state of insensibility, from which he recovered, though the pupil of the right eye continued preternaturally dilated. A middle-aged man broke his arm, and received in addition, compound fracture, with slight depression of the left parietal bone. The only symptom that was noticed was dilatation of the right pupil, but during the process of shaving the head, he was seized with delirium. He was bled with relief—relapsed again—and was a second time bled with advantage. Remaining insensible, the trephine was applied, but although he experienced a return of sensibility, stupor supervened, and terminated fatally. A disjunction of the coronal suture was discovered—more than a drachm of blood extravasated between the dura mater, on the right side, and frontal and parietal bones—a partial and slight extravasation between the tunica arachnoides and pia mater—and lastly, a small quantity of pus both over the dura mater, and between the arachnoid and pia mater. In another case, delirium was followed by stupor, which was relieved by the abstraction of twenty ounces of blood, but again re-appeared. One pupil was dilated, but the patient recovered. In this case, there was probably pressure on the brain, and it seems that delirium, like convulsions, is produced by such pressure as acts as a source of irritation, without being so extensive as completely to annihilate the functions of the organ.

We now arrive at the subject of treatment, and first of the

### VI.—TREATMENT OF CONCUSSION OF THE BRAIN.

This occupies the 7th Section of the paper, and is laid down in a clear and exceedingly interesting manner. How important it is that a rational and scientific plan of treatment should be inculcated, and generally pursued, we need scarce inform those who are acquainted, as most men must be, with the doubts, the disputes, and the blunders that are daily and disgracefully occurring.

In the treatment of concussion we are required, as Mr. Brodie observes, to medicate not only for the present but the future—to contribute to the relief of symptoms that exist, and mitigate or prevent the occurrence of after ones. The profession, or at least a good part of it, would seem to be split into two distinct sects, the one recommending the exhibition of stimulants and cordials, the other antiphlogistics and blood-letting. Here, however, as elsewhere, Mr. Brodie believes that the difference of opinion is greater in appearance than reality.

"I suppose that none of those who have suggested the exhibition of stimulants would actually be inclined to apply this practice to cases in which the pulse has regained its strength and regularity; and, on the other hand, I conclude that no one among those who have advised the use of the lancet would think of taking away blood when the patient lies with pale cheeks, and cold extremities, and a feeble and intermitting pulse, or would refuse to resort to the cautious exhibition of cordials and stimulants where these symptoms are so urgent that he is manifestly in danger of sinking, in consequence of the depressed state of the circulation which has followed the first shock of the injury." 53.

Mr. Brodie, we fear, has let his good nature prevail over justice, in the latter part of the preceding quotation. Cases are, we will not say commonly, but certainly not *very* unfrequently occurring, where patients are bled, or attempted to be bled, with a pulse that can scarcely be felt, and a surface as blanched as a surface may be!

Cases of dangerous depression of the powers are in reality of very rare occurrence, a condition of the system approaching to syncope being generally salutary, and preventing the effusion of blood from the smaller vessels which are frequently ruptured by the blow that produces what we term the concussion. By exhibiting stimulants we endanger this effusion, and consequent symptoms of pressure on the brain, whilst keeping down the action of the heart, by bleeding on the rise of the pulse to a sufficient amount, will probably often succeed in arresting it entirely, or checking what might otherwise be fatal. We should also bear in mind, that depression is followed by excitement; the pulse, as the patient recovers, growing fuller and stronger than natural, and affording an additional argument in favour of the practice recommended above. Whilst we carefully guard against present extravasation, we should also remember that secondary hæmorrhage, a case of which has already been detailed, may possibly occur within the cranium. However small the chance, says Mr. B., the surgeon should provide against it by frequently inquiring into the condition of his patient, by enjoining repose both of body and mind, by a scanty and vegetable diet, the exhibition of laxatives, and abstraction of blood whenever it is warranted by the state of the pulse. Whilst Mr. Brodie is of opinion that the patient would generally recover spontaneously from the state of insensibility into which he is plunged, still there cannot be a doubt that recovery is assisted by low diet, repose, and depletion. Besides, inflammation of the brain, or its membranes, is surely less likely to occur where depletion has been carried to a *moderate extent*, than when it has been totally neglected. We have said the depletion should be moderate, for Mr. B. remarks that the surgeon may err in employing the lancet too freely. If very large quantities of blood are abstracted, and inflammation is afterwards developed, the hands of the practitioner are tied, his resources are crippled, and, to use the expression of an eminent surgeon, the patient can neither support the disease nor the doctor! Mr. Brodie recommends that at first when reaction has occurred, the patient should lose such a quantity of blood as completely to subdue the force of the circulation, but that afterwards, the bleeding be occasional only, and then to a moderate extent. The following is important.

"Where bleeding has been carried to a great extent, symptoms frequently occur which in reality arise from the loss of blood; but which a superficial observer will be led to attribute to the injury itself, and concerning which indeed it is sometimes difficult, even for the most experienced surgeon, to pronounce in the first instance to which of these two causes they are to be referred. Repeated copious blood-letting is of itself adequate to produce a hardness of the pulse, which we shall in vain endeavour to subdue by persevering in the same system of treatment. In many individuals it will produce head-ach and confusion of mind, not very different from what the injury itself had previously occasioned. These things may be observed especially in young females who are disposed to hysteria; and whom I have often known to suffer from a continued aggravation of such symptoms as I have described, while the system of depletion has been continued, recovering immediately on the use of the lancet being laid aside, and on their being allowed to take solid nourishment, with occasional doses of the carbonate of ammonia." * p. 58.

In all cases of injury of the brain, the head and shoulders should be raised by additional pillows.† In bad cases the head should be shaved, and cold lotion constantly applied. Opiates are injurious, as they constipate the bowels, and occasion a head-ache of which it is difficult to determine, whether it belongs to the injury or opium.

### SECT. VII.—TREATMENT OF COMPRESSION OF THE BRAIN.

This is necessarily more complicated than that of concussion, and does not admit of such simple or general rules. This section is dedicated to the subject of pressure on the brain, without any wound of the dura mater. Mr. Brodie sets out with the general principle, that where the symptoms of compression are such that the life of the patient is in danger, the cause should unquestionably be removed, where that can be done, by a surgical operation. The most common cause of pressure on the brain, is extravasation of blood *within* the cavity of the dura mater, to the consideration of which we shall first proceed, although Mr. Brodie adopts a different arrangement. If there exist only one large collection, it is mostly at the basis of the cranium, sometimes in the substance of the brrain, sometimes betwixt the arachnoid and pia mater, in any case beyond the reach of operations. As a general rule, operations are inapplicable to cases of internal extravasation, but what rule is so general, after all, as never to admit of exceptions? Extravasation may occur upon the surface of the cerebrum, immediately under the dura mater, but what are its symptoms? how can we detect its existence? The man who can answer this question is a cunning or confident diagnost indeed! Suppose, however, that the bone is already removed, and the dura mater exposed, of a blueish tint, and bulging into the aperture, from the pressure of extravasated blood beneath it. Wounds of this membrane are exceedingly dangerous, and should never be "wantonly" performed; but desperate cases admit either of desperate remedies or none, and the patient, one would think, has a right to that chance, minute as it may be, which division of the dura mater gives him. The combination of circumstances is necessarily rare, but still it *may* occur, and the surgeon *must* meet it.

The late Mr. Chevalier was called to a child, who, after receiving a blow upon the head, became insensible, and affected with convulsions. The scalp

---

* Dr. Marshall Hall has published, in the thirteenth volume of the Medico-Chirurgical Transactions, some excellent practical observations on the effects of copious blood-letting, many of which are applicable to the cases mentioned above.

† The "air pillows," so much in vogue at present, are convenient.—REV.

was not wounded, but the fontanel appearing to be elevated, M. Chevalier made a crucial incision of the scalp, divided the fontanel by an angular incision on its right side, and, finding the dura mater raised by extravasated blood, cautiously punctured the membrane with a lancet, when the blood spouted out to the distance of a foot. The symptoms were relieved, and the child recovered. A still more remarkable case was communicated to Mr. Brodie, by Mr. Ogle, of Great Russel-street. Having previously related this case, we shall give but a very brief notice of it here. A woman, after receiving a fall upon her head, was found by Mr. O. in a state like that of apoplexy. No wound of the scalp was discovered, but pressure near the anterior superior angle of one of the parietal bones made her flinch very much. The scalp at this spot was divided—no fracture appeared—but the danger being imminent, a portion of bone was removed with the trephine, when the dura mater immediately bulged into the opening, and presented to the fingers the feeling of fluid beneath it. The dura mater was punctured—a jet of blood spirted out to the height of some feet, and the patient, who had hitherto been totally insensible, immediately recovered her senses, and never had a bad symptom afterwards. These cases must be considered Oases in the desert, and quite "out of the common course of events." Mr. Brodie recommends their being treated like apoplexy, by bleeding from the arm, cupping, saline purges, the local application of cold, and other means, which, though palliative only, will be followed by recovery in many cases where the symptoms of pressure have been urgent. In some instances, the patient recovers, though slowly; in others, there may be partial paralysis—dilatation of a pupil, or some such memento of the accident. So much for extravasation internal to the dura mater.

When the hæmorrhage takes place between the dura mater and bone, an operation is required.

"But here another question arises: what is the evidence which is to enable us to detect a mass of extravasated blood in this situation, and how are we to determine what is the exact part of the cranium which should be perforated by the trephine? I must here refer to an observation which has been already made. Blood is seldom poured out in any considerable quantity between the dura mater and the bone, except in consequence of a laceration of the middle meningeal artery, or one of its principal branches, and it is very rare for this accident to occur, except as a consequence of fracture. If, therefore we find the patient lying in a state of stupor, and on examining the head we discover a fracture, with or without depression, extending in the direction of the middle meningeal artery, although the existence of an extravasation on the surface of the dura mater is not thereby reduced to an absolute certainty, it is rendered highly probable, and the surgeon under these circumstances would neglect his duty if he omitted to apply the trephine." 61.

If any extravasation is found, the present symptoms are relieved, and the chance of the patient's recovery increased, whilst, if none should exist, his condition can scarcely be worse than before. Where no fracture can be found, if the injury has been inflicted in the situation of the meningea media, the use of the trephine is a fair speculation, sufficiently justified by the instances on record of rupture of the artery without any fracture, as well as by the fact, that the inner table may be broken independent of fracture of the outer. The observation of Mr. Abernethy, that the bone will not bleed with its natural freedom and celerity, when blood is extensively effused between it and the dura mater, may possibly assist our judgment. This, however, is a test on which we should rely very little, and, indeed, at the best, it can only apply when the surgeon is already half through his operation.

In applying the trephine for fracture with depression, the removal of a small portion of bone is in general sufficient, but not so in cases of extravasation on the surface of the dura mater. Mr. Brodie was led to this conclusion by a case which occurred to him, where he removed two triangular pieces of bone, and gave vent to a quantity of blood, partly fluid, partly not. Suppuration ensued on the surface of the dura mater, wherever the extravasation had formerly been; the opening in the bone became mostly filled up by granulations; the pus, which was, in consequence, confined, burrowed between and separated the dura mater and bone, and although another portion of the latter was removed, it was followed by scarcely a temporary relief, and the patient unfortunately died. Mr. Brodie believes that if the opening in the bone had been freer in the first instance, the pus which was formed would have found a ready exit, and the patient have probably recovered.

It is very well known that fractures, with extensive depression, may exist, without a corresponding severity of symptoms, or even without any symptoms whatever. An interesting and intricate question arises, as to whether the surgeon should operate or not, a question which is answered in a very different manner by Mr. Abernethy and Sir A. Cooper! In the first place, the removal of part of the cranium is not to be considered a mere *bagatelle*, warranted on slight grounds or no grounds at all. Where the aperture is small, it requires many years to fill it up with new bone, and, where large, the deficiency is never supplied. The defence of the skull-cap is consequently weaker—the cicatrix more readily penetrated or torn—more likely to suffer from the force of concussion than the original bone that was removed. A case is recorded in the Edinburgh Medical Essays, where a child, in a violent fit of the hooping-cough, burst the cicatrix, when the dura mater also gave way, the brain was protruded through the wound, and death was the consequence in the course of five days. 2dly. The operation of the trephine is occasionally followed by sloughing of the dura mater, though previously perfectly healthy. Mr. Brodie has witnessed two cases of this kind, and concludes that the sloughing of the membrane is the consequence of its being deprived of its natural protection, as well as of the loss of the blood it receives through the vessels of the bone.

"If the patient had survived some time longer, what would have happened? The slough of the dura mater would have separated, and the brain, losing the support which it derives from this firm membrane, and having its vessels loaded with blood would, in all probability, have become protruded in the form of what is denominated a *hernia cerebri.* Such a protrusion would not indeed aggravate the danger of the case, where suppuration had already taken place within, but it might make the difference of life or death to the patient where the inflammation had not begun to terminate in this manner." 72.

The cases recorded by Mr. Stanley, in the 8th volume of the Medico-Chirurgical Transactions, satisfactorily prove that hernia cerebri is liable to follow the mere abstraction of a portion of the cranium. From these considerations Mr. Brodie thinks it obvious, that the operation of trephining is attended in itself with some degree of danger. These are the *cons.* let us now look a little at the *pros.* 1st. Although sloughing of the dura mater and protrusion of the brain are occasional, they cannot be considered as constant sequelæ of the operation of the trephine. 2dly. Notwithstanding depression of the cranium is sometimes unattended with mischief, there are numerous examples to the contrary. Suppuration ensues on the surface of the dura mater, and, ultimately, the matter has no free discharge, the inflammation extends to the arachnoid and pia mater, and speedily ends in the patient's destruction. 3dly. A depression may occasion no mischief at first, but, after a certain lapse of time, be followed by symptoms endangering even the life of the patient. A good many cases of this kind are recorded, one or two, we believe, by Sir A. Cooper, in his Surgical Lectures. Mr. Brodie alludes to the case of a gentleman, published by Sir Everard Home, in the Philosophical Transactions for 1814. The depression was, in its longest diameter, two inches and a quarter, and an inch and a half in its shortest, and in one part nearly three quarters of an inch below the natural level. At the end of six weeks the early symptoms had subsided, but, as soon as he returned to his usual occupations, various nervous phenomena shewed themselves, which, instead of diminishing, increased in severity, and led Sir E., at the end of three years, to remove nearly the whole of the depression by means of the trephine. The symptoms were relieved, and returned no more. Mr. Brodie believes that these ultimate ill-consequences might perhaps be avoided, if the rule were observed, to trephine where the depression is deep or extensive. This rule is inapplicable, of course, to cases of suppuration between the bone and the dura mater, which latter is as likely to occur where the depression is small as where it is large.

The majority of our readers must be very well aware that Sir A. Cooper, in his Lectures, has drawn a distinction between simple and compound fractures of the skull; inflammation and suppuration of the membranes of the brain being infinitely more liable, according to the Baronet, to occur in the latter variety of injury than the former. In compound fracture with depression, Sir Astley immediately applies the trephine—in simple, he does not, unless, indeed, the symptoms imperiously demand it. Though many individuals recover in whom the more serious injury existed, and for which no operation whatever was performed,* the question, nevertheless, is not to be decided in so hasty and sum-

* The following statement, furnished to Mr. Brodie by his friend Mr. Rose, is strongly illustrative of this.

"In the battle of Talavera de la Reyna, which was fought on the 27th and 28th of July 1809, the brigade of guards lost about 600 men in killed and wounded. Amongst the latter were a considerable number of cases of wounds in the head. There were a great many cases of fracture of the bones of the cranium with and without depression, and from the cause which produced them, these were, of course, in every instance complicated with wounds of the scalp.

mary a manner. The science of medicine is a science of conjecture, and that plan of treatment is accounted the best which has merely the balance of chances in its favour. If, then, it shall be found from the results of experience, that a greater probability exists of suppuration occurring in those cases of fracture where the scalp is wounded also, than where it is not, Sir Astley's opinion is substantially correct. Mr. Brodie at first was inclined to dispute it, but, on looking over notes which were taken by himself at the bed-side of patients, for the most part before this question had been agitated, he finds that the cases in which suppuration takes place *where the scalp is entire*, have been comparatively rare; bearing but a very small proportion to those where it has succeeded a fracture with scalp-wounds. From attentively considering the whole of the evidence, Mr. Brodie acknowledges that it now appears to him that the views of Sir Astley Cooper are correct, and that, in those cases where depression of bone exists without any symptoms, or trifling ones only, the surgeon can follow no better rule than this;—trephine if it be combined with a wound of the scalp, but if such a wound is not present, let him not make it by a surgical operation. If the cranial depression is extensive, perhaps it would be better to elevate it at all events, not because the danger of suppuration is greater, but on account of the ill-effects which the patient may ultimately experience, if the brain be left subjected to considerable pressure.

A remark of much importance is made by Mr. Brodie, viz. that in cases of compound fracture, the bone may be depressed in such a manner, as to allow of the escape of any matter which may form, although the depression remain. If the abscess can find no outlet whatever, its danger must be infinitely greater. In the case of a boy with compound fracture of the skull, the symptoms were so slight that the trephine was not applied. Eight or nine days after the accident, the pulse being frequent, the countenance anxious, and the patient complaining of pain in the head, the fracture was completely exposed by an incision. Suppuration had occurred beneath the bone, but its edge was so placed, that a very free opening remained for the pus. No further operation was, therefore, performed—the symptoms were relieved—and the patient very shortly recovered. In cases where the scalp remains entire, but the brain and its membranes are wounded by the edges of the fracture and depression, there is reason to suppose that more or less suppuration must necessarily take place, if the parts are exposed by incision of the scalp, while it seems not improbable

---

"'On the 3d of August, in consequence of some military movements, the town of Talavera, in which the hospital had been formed, became exposed, and an order was given for all the wounded who could march, to leave it. This was so speedily obeyed that no time was afforded to make any selection. The worst cases necessarily remained, but among those who undertook the march there were twelve or fourteen with wounds in the head, accompanied with injuries of the bone, at least four or five of whom had both tables of the skull fractured, and two of them, along with fracture of the os frontis, had each the globe of one eye totally destroyed. In none of them had the trephine been applied, nor had any attempt been made to remove splinters of bone. After leaving Talavera, they were exposed to a burning sun, and to very severe fatigue. Every evening, after the day's march, Mr. Rose collected the wounded round him, examined and washed their wounds, dressing with care those that particularly required it. Cold water was the principal application employed. The retreat occupied sixteen days, in spite of which, and with no other treatment than that which has been described, every one of those who were wounded in the head recovered.'"

that the mischief may be avoided, if this remain entire for their covering and protection. This is an additional inducement to leave the scalp intact in cases of fracture and depression of the cranium.

Sect. VIII.—Treatment of Contusions and Wounds of the Scalp.

Extravasation of blood in the cellular texture of the scalp is sometimes extensive, but mostly requires no particular attention, the swelling spontaneously and gradually diminishing, and in no great length of time disappearing altogether. In the case of a young gentleman, the effusion extended all over the head, from eyebrow to occiput, and ear to ear, but entirely subsided in the course of a few weeks, with no other application than that of cold lotion. In another case which threatened to become as extensive, pressure at the point where the extravasation had begun succeeded in putting a stop to its progress. A child struck its temple, soon after which a swelling appeared, and increased on the following day. Mr. Brodie directed the child to be kept quiet, and ordered the head to be bathed with cold lotion. Next day the swelling having greatly extended, Mr. Brodie ascertained the exact spot which had been struck, and applied such a graduated compress and bandage, as is used after bleeding in the temporal artery. The swelling was by this means totally arrested. Wounds of the scalp, whether punctured or incised, require, in the first instance, no particular treatment. Mr. B. never witnessed injurious effects from the employment of sticking-plaster in wounds of the scalp. Flaps of the scalp, when formed by a wound, should be carefully replaced, and unless the exposure has been long, the contusion considerable, or the surface smeared over with dirt, they generally unite by the first intention. Sometimes the adhesions are partial, suppuration occurring elsewhere, and requiring much care on the part of the surgeon, to obviate injury to the bone or pericranium. In cases where the pericranium is detached, the scalp should be also replaced for the chance of the union which frequently takes place in adults, and oftener still in young persons, on account of the greater vascularity of the textures.

Sect. IX.—Treatment of Fractures of the Cranium, unattended with Depression.

The general opinion in cases of fracture, unattended with depression or evidence of any considerable extravasation between the dura mater and bone, is not to employ the trephine, but only those strict antiphlogistic measures required in all cases of injury of the head. The fractured surfaces being here placed in contact are favourably disposed to unite with each other, whilst removal of a portion of the bone of the cranium is a wanton and ridiculous addition to the mischief. A great part of the section is occupied by Mr. B. in combating the views of Mr. Pott on this subject, but as we believe they are now universally abandoned, we shall content ourselves with stating the general principle; that in fracture of the cranium, unaccompanied by depression, the active interference of art is not required. These cases, however, Mr. Brodie remarks, should always be regarded with a jealous eye, as a danger exists of the formation of matter on the dura mater, especially when the scalp is also wounded and the pericranium separated from the bone. We now pass on to,—

### Sect. X.—Treatment of Wounds of the Brain and its Membranes.

Though concussion or compression with this complication is essentially more dangerous than without it, the general treatment is nearly the same—bleeding, purgation, starvation, and repose. Our local means must be mainly directed to prevention of future ill-consequences, the chief of which are—inflammation extending from the wound to the membranes, and producing effusion of serum and pus—inflammation, suppuration, sloughing, and dissolution of the substance of the brain—and, lastly, protrusion of the brain in the form of a hernia cerebri. Mr. Brodie insists on avoiding, as much as possible, meddling with the parts. If splinters of bone have penetrated the brain, and admit of being readily extracted without any additional disturbance of the organ, such extraction "cannot be improper, and may probably be useful." Many have, however, recovered where the splinters have remained, an instance of which occurred to Mr. Brodie, detached fragments of bone continuing imbedded in the brain of a gentleman, and causing no disturbance, many months after the reception of the injury.

"Do not such cases justify us in leaving splinters of bone untouched, where there is any kind of obstacle to their easy extraction? Are they not even sufficient to show that any other mode of proceeding would be improper, and that it is better to leave the patient to take his chance with the splinters lodged in the brain, than to commit the smallest additional violence in an endeavour to remove them?" 90.

The principle holds in wounds of the brain from depression of bone. If the edge of the bone can be readily raised by the elevator or forceps, it may be right to do so; but persons recovering, in whom the depresion is allowed to remain, it cannot be advisable to hazard this chance whatever it may be, if the elevation requires such force "as is likely to cause the most trifling additional injury to the wounded brain." Where no circumstances exist to render the operation absolutely necessary, Mr. Brodie is inclined to doubt its expediency, as the motion of the saw, of little consequence, perhaps, where the membranes are entire, must jar more or less the structure of the brain, when lacerated already and contused. The liability to the occurrence of hernia cerebri is here, as elsewhere, an objection to the removal of any considerable portion of the cranium.

If a foreign body lodged in the brain possesses such form and dimensions, that one of its extremities projects externally, it may be easily removed, and probably ought to be extracted at all risks. If the body, however, be a bullet or ball the case is very different. In the first place, the ball can be seldom discovered and extracted without very serious or even fatal violence; and, secondly, numerous instances exist, where patients have survived, though the ball was allowed to remain. Some of these, indeed, have suffered no more than they probably would have done, if the ball had been lodged in any unimportant part of the body. On these accounts, Mr. Brodie proposes, as a general rule, that the extraction of a ball should not be attempted, unless it can be done without the employment of violence, or adding, in any degree, to the mischief already inflicted. On the whole, Mr. Brodie believes that negative is better than active local treatment in most of the cases of wounds of the brain. The proportion of cases that occur in civil hospitals is generally small, the recoveries under every circumstance so few, that no one can determine, from his own experience merely, the comparative value of this or that treatment. The safest plan to follow, when deserted by art, is to trust as much as may be to Nature. The following quotation will be read with interest.

"Where the experience of individuals fails, we are called on to look for other sources of information. I have referred to all the cases of wounded brain recorded in the works quoted below,* and the general results which they exhibit will be found not uninteresting, if viewed in their relation to this point of surgical practice. These cases are thirty-eight in number, of which twenty-six terminated favourably, and twelve unfavourably. This, of course, affords no information as to the actual rate of mortality in cases of this description, the fatal cases being for the most part regarded as too much a matter of course to be worthy of publication, while a very different opinion is entertained respecting the cases of recovery. But the following facts afford some useful information as to the circumstances under which recovery takes place.

"In nine cases of wounded brain in which the bone was fractured, but not depressed, no operation whatever was performed. In two of them the patients died; in the remaining seven they recovered.

"In fifteen cases no operation was performed, beyond that of removing some splinters of bone with the forceps. In five of these cases the patients died, while in ten the patients recovered.

"In four cases the wound of the brain was complicated not only with fracture, but with depression of bone. In one of them in which the depressed bone was allowed to remain without being elevated, the patient recovered. In the three remaining cases the depression was elevated with the assistance of the trephine; and one of these patients recovered, and two of them died.

"In ten cases a musket ball was lodged in the brain. In two of them the ball was extracted, and one patient recovered, while the other died. In the remaining eight cases the ball was allowed to remain, no attempt being made for its extraction, and two of these patients died, while six of them recovered. Of these last, however, one died several weeks afterwards of inflammation of the brain induced by intemperance in drinking, and another, after having been sufficiently well to resume his duties as a soldier, died in the course of the following year, of what was regarded as a *coup de soleil*.

"It appears then that in fourteen out of twenty-six patients who recovered, no operation whatever was resorted to, and that in ten of the remaining twelve, there was no operation beyond that of removing splinters of bone with the forceps. Of those in whom a ball was extracted from the brain one died, and one recovered; and of those in whom the ball was not extracted two died, and six recovered. It is needless to add, that the conclusions to be deduced from these statements illustrate and confirm the observations which have been already made as to the principles which should direct the surgeon in his treatment of these formidable injuries."†

Mr. Brodie has never been able to discover an instance of recovery from a wound of the posterior lobes of the cerebrum, the cerebellum, or medulla oblongata. In most of the cases the injury was confined to the frontal bone, and that part of the brain which it covers and defends. This is important in assisting, in some measure, the surgeon's diagnosis.

---

* "Mémoires de l'Académie Royale de Chirurgie.—Transactions of a Society for the Improvement of Medical and Chirurgical Knowledge.—Duncan's Medical Commentaries.—Duncan's Annals of Medicine.—Edinburgh Medical Journal.—Medico-Chirurgical Transactions, Vol. I. to Vol. XII. inclusive.—Le Dran's Observations in Surgery.—Hennen's Military Surgery.—Collection d'Observations Cliniques par M. A. Petit."

† "Since these calculations were made, a very interesting case has been published by Dr. Rogers in the thirteenth volume of the Medico-Chirurgical Transac-

### SECT. XI.

This section concludes the inquiry, and is dedicated to "the treatment of some other cases, not included under the foregoing heads."

In cases of loss of a particular sense, as that of smell, no remedies are indicated beyond what are adapted to ordinary cases of concussion of the brain. The cure, when it occurs, being not so much owing to the skill of the surgeon as the powers of nature. Sometimes the function is permanently lost. When a patient is affected with furious delirium, blood should immediately be taken from the arm, in a full stream if possible. The delirium almost invariably yields, although a relapse may render it necessary to resort to venesection a second or third time, before any permanent relief is obtained. Convulsions occurring under circumstances similar to those of delirium, would seem to require a similar treatment. This though in part is not wholly the case; for bleeding, though indicated in convulsions which immediately follow an accident, is sometimes incapable of arresting them when fairly established. In many cases they continue, notwithstanding the loss of a quantity of blood, subsiding spontaneously at last. Occasionally they occur at the end of some days, it may be, combined with inflammatory symptoms, and require the further employment of the lancet. They may, however, exist without inflammation, being aggravated by bleeding, and only disappearing when the patient is allowed some substantial nourishment. A case of this kind has already been detailed.

Our analytical labours having drawn to a close, we have little to add in the shape of observation. The task of the critic, or rather "analytic," though frequently unpleasant, and always laborious, is useful alike to the individual and the public. The former must pick up a pretty intimate acquaintance with the practice and precepts of his author, and the latter is presented, at a moderate expense, with the gist and the marrow of both. The critic is, in fact, a kind of jack-all to the public, or rather, resembles the minister attached to the despot of old, whose office consisted in chewing his food and rendering it fit for, and easy of digestion! To return to the subject of the present analysis, we think it must be obvious to all who are actually engaged in their profession, how interesting and important a topic it is. The discrepancies of opinion in the minds of the profession, their differences, and, frequently, blunders in treatment, imperiously required a systematic view of the various and complicated injuries of the head, a view which should be practical, founded on experience and illustrated by it. We firmly believe, and we freely avow it, that Mr. Brodie's paper is eminently qualified to answer these desirable ends. Unbiassed by theory, untainted by prejudice, the author has detailed the results of his practice—acknowledged his errors where he thought he had committed them

---

tions, in which the breech-pin of a gun was lodged in the anterior lobes of the cerebrum, and extracted at the end of twenty-eight days, the patient afterwards recovering. Here the foreign body remained in the substance of the brain until inflammation had been going on for an entire month, so that the brain and its membranes must have become extensively agglutinated and consolidated around it. The question as to the extraction of a foreign body after such a lapse of time, and under such circumstances, belongs more properly to another part of these inquiries; my observation at present being intended to be confined (as nearly as that can be done) to the treatment to be employed immediately or soon after the occurrence of the injury."

—and placed upon record a body of *facts*, which must ever be of value to the practical man, although unadorned with the flimsy decorations,

Of florid prose, or honied lies of rhyme.

We had finished this article, and were mightily pleased with the result of our labours, when the following damper was put into our hands.

"*Throughout the one hundred pages which are devoted to Mr. Brodie's* '*Pathological and Surgical Observations,*' *there is not a single line worthy of transcribing. The paper is made up of the most common-place, familiar to the merest tyro, and to be found in every elementary work on surgery.*" The reviewer accordingly shirks it altogether, and occupies his page with the case of a patient, who sweated so hard, that he flooded the clinical ward of Guy's Hospital! Seriously, was ever such barefaced partiality, such wanton dereliction of literary honesty, on the part of a journal professing to cater for the public instruction? Because an individual is at the head of his profession, and *known* to be hostile to the party which disgraces it, the dagger of malignity is aimed at him and his, whilst any petty lecturer who stoops to curry favour with the vilest of presses, is lauded to the skies with its dirty panegyric. Verily, the labourer is worthy of his hire, a hire which is worse indeed than worthless. The censure of a journal like this is inestimable—its praise to the possessor, a fief of degradation, held by the tenure of the basest apostacy!

P.S. So much for the Review of Mr. Brodie's paper. Let us now see the *sham* analysis of that of Mr. Lawrence. This Analytical Review consists of five short extracts, in *ipsissimis verbis* of Mr. Lawrence, without any intervening connexion by the reviewer, and occupying *four pages and three quarters* of the Lancet! Having strung these extracts together, without literary needle or thread, the modest reviewer placidly adds:—"In giving this summary (!!!) of Mr. Lawrence's paper, *we have carefully embraced* all *the leading points; so that our readers may consider themselves in possession of a* concise statement *of the talented author's views and practice in erysipelas!!*" Now as this "concise statement" is in Mr. Lawrence's own words, and occupies less than five pages, we ask Mr. Lawrence how he could impose so heavy a tax on the time and pockets of his professional brethren, as to dilate his paper to the immense extent of 221 octavo pages? The *imposture* and falsehood set forth in this mock review will be apparent to all the better classes of readers; but they will go down quite smoothly with the ignorant apprentices of chemists, druggists, and hedge-accoucheurs, who form the principal readers of the journal in question.

But even so short an article as the summary of Mr. Lawrence's paper cannot be put forth without a specimen of ignorance and misrepresentation. The Lancet is in a rage with Mr. Lawrence for descending into the arena with such insignificant personages as one Henry Earle, and a "Mr. Arnold, Surgeon of the Grampus!" Whether our readers may have ever heard of Mr. Earle, we cannot pretend to say—but the poor Surgeon of the Grampus has little to answer for in this literary contest. The individual alluded to is Mr. Arnott, Librarian to the Medico-Chirurgical Society, whose ship, the Grampus, rides quietly at her moorings in New Burlington Street.

That we have done literary justice to the production of Mr. Lawrence and Mr. Brodie, uninfluenced by party spirit, the present number of our Journal will amply prove. We can conscientiously say, what every reviewer should be able to say—

Tros Tyriusve mihi nullo discrimine agetur.
